# Evodiamine Induces Apoptosis in SMMC-7721 and HepG2 Cells by Suppressing NOD1 Signal Pathway

**DOI:** 10.3390/ijms19113419

**Published:** 2018-10-31

**Authors:** Xing-Xian Guo, Xiao-Peng Li, Peng Zhou, Dan-Yang Li, Xiao-Ting Lyu, Yi Chen, Yan-Wei Lyu, Kuan Tian, De-Zhi Yuan, Jian-Hua Ran, Di-Long Chen, Rong Jiang, Jing Li

**Affiliations:** 1 Lab of Stem Cell and Tissue Engineering, Department of Histology and Embryology, Chongqing Medical University, Chongqing 400016, China; 17774968247@163.com (X.-X.G.); 17358487423@163.com (X.-P.L.); zp979391906@163.com (P.Z.); ldztd@163.com (D.-Y.L.); sungirlxt@163.com (X.-T.L.); chenyi6810@126.com (Y.C.); happyeveryday.1114@163.com (Y.-W.L.); xinmengyuandl@163.com (D.-L.C.); 17709170419@163.com (R.J.); 2 Neuroscience Research Center, College of basic medicine, Chongqing Medical University, Chongqing 400016, China; tkcomeon@126.com (K.T.); yuandezhi33@yeah.net (D.-Z.Y.); ranjianhua2013@163.com (J.-H.R.); 3 Chongqing Three Gorges Medical College, Chongqing 400016, China

**Keywords:** evodiamine, hepatocellular cancer, NOD1, NF-κB, MAPK, apoptosis

## Abstract

Hepatocellular cancer (HCC) is a lethal malignancy with poor prognosis and easy recurrence. There are few agents with minor toxic side effects that can be used for treatment of HCC. Evodiamine (Evo), one of the major bioactive components derived from fructus *Evodiae*, has long been shown to exert anti-hepatocellular carcinoma activity by suppressing activation of nuclear factor-κB (NF-κB) and mitogen-activated protein kinase (MAPK). In addition, in the Nucleotide-Binding Oligomerization Domain 1 (NOD1) pathway, NOD1 could initiate NF-κB-dependent and MAPK-dependent gene transcription. Recent experimental studies reported that the NOD1 pathway was related to controlling development of various tumors. Here we hypothesize that Evo exerts anti-hepatocellular carcinoma activity by inhibiting NOD1 to suppress NF-κB and MAPK activation. Therefore, we proved the anti-hepatocellular carcinoma activity of Evo on HCC cells and detected the effect of Evo on the NOD1 pathway. We found that Evo significantly induced cell cycle arrest at the G2/M phase, upregulated P53 and Bcl-2 associated X proteins (Bax) proteins, and downregulated B-cell lymphoma-2 (Bcl-2), cyclinB1, and cdc2 proteins in HCC cells. In addition, Evo reduced levels of NOD1, p-P65, p-ERK, p-p38, and p-JNK, where the level of IκBα of HCC cells increased. Furthermore, NOD1 agonist γ-D-Glu-mDAP (IE-DAP) treatment weakened the effect of Evo on suppression of NF-κB and MAPK activation and cellular proliferation of HCC. In an in vivo subcutaneous xenograft model, Evo also exhibited excellent tumor inhibitory effects via the NOD1 signal pathway. Our results demonstrate that Evo could induce apoptosis remarkably and the inhibitory effect of Evo on HCC cells may be through suppressing the NOD1 signal pathway in vitro and in vivo.

## 1. Introduction

With 854,000 incident cases diagnosed and 810,000 deaths globally in 2015, hepatocellular cancer (HCC) is the most common primary malignancy of the liver and is the third leading cause of cancer-related death worldwide [1,2]. It is characterized by high morbidity and mortality, high risk of intrahepatic recurrence, and poor prognosis [3,4,5]. Moreover, there are some problems with leading conventional chemotherapeutic agents such as poor targeting efficiency, strong adverse effects, and poor tolerance [6,7]. Recently, studies have suggested that a variety of natural products can exhibit anti-cancer activity while displaying low toxicity [8,9].

Evodiamine (Evo), an active ingredient isolated from the fruit of *Evodia rutaecarpa* Bentham, has diverse well-defined biological activities including anti-obesity, anti-inflammation, and anti-tumor effects [10,11,12,13,14]. Researchers have found that Evo can induce apoptosis in human ovarian cells, lung carcinogenesis, pancreatic cancer, breast cancer, human melanoma cancer, thyroid cancer, human bladder cancer, human leukemia, colon cancer, and hepatocellular cancer [15,16,17,18,19,20,21,22,23,24]. Among them, antitumor activities through the suppression of nuclear factor-κB (NF-κB) and mitogen-activated protein kinase (MAPK) activation have been extensively investigated [15,20,25]. Although this suppression by Evo on HCC has been known for several years [17], molecular details that underline this process are still being uncovered.

Furthermore, it has been reported that in the Nucleotide-Binding Oligomerization Domain (NOD1) pathway, NOD1 could initiate NF-κB-dependent and MAPK-dependent gene transcription [26]. The NOD1 pathway is expressed in most tissues, including cancer cells. Researchers have gathered data of NOD1 levels in the GEO database and revealed that NOD1 expression differed significantly between tumor and non-tumor tissue [26]. In addition, recent experimental studies reported that the NOD1 pathway was related to controlling development of breast [27], head and neck squamous cell carcinoma [28], gastric carcinoma [29], and lung cancer [30]. Therefore, we hypothesize that Evo exerts anti-hepatocellular carcinoma activity by inhibiting NOD1 to suppress NF-κB and MAPK activation.

In this study, to determine the function of Evo in controlling growth of HCC and the effect of Evo on the NOD1 signal pathway, we proved the effect of Evo on proliferation of HCC cells and detected changes in the NOD1 pathway in vitro and in vivo. When treated with Evo, the cell cycle significantly was arrested at G2/M phase, P53 and Bax proteins were upregulated, and B-cell lymphoma-2 (Bcl-2), cyclinB1, and cdc2 proteins were downregulated. Additionally, levels of NOD1, p-P65, p-ERK, p-p38, and p-JNK were reduced and the level of IκBα was increased. Furthermore, NOD1 agonist γ-D-Glu-mDAP (IE-DAP) treatment weakened the effect of Evo on suppression of NF-κB and MAPK activation and cellular proliferation of HCC. Our results demonstrate that Evo could induce apoptosis remarkably and the inhibitory effect of Evo on HCC cells may be through suppressing the NOD1 signal pathway in vitro and in vivo.

## 2. Results

### 2.1. Evo Inhibits Cell Viability and Induces Cell Apoptosis in HCC Cells In Vitro

**Figure 1:**
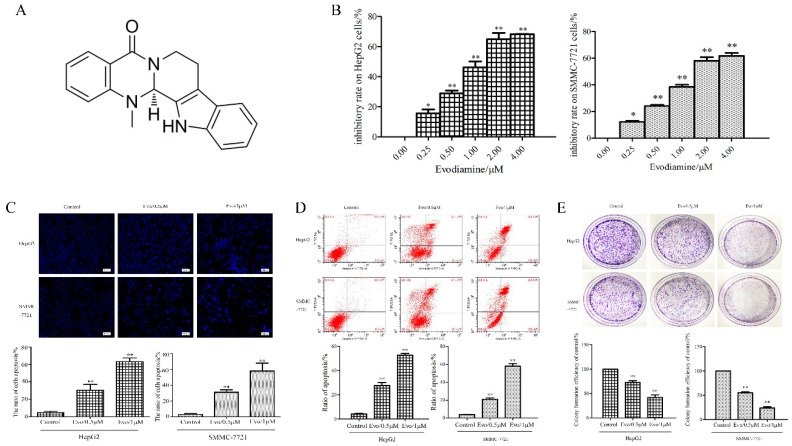
Evodiamine (Evo) inhibits cell viability and induces cell apoptosis in hepatocellular cancer (HCC) cells in vitro. (**A**) Chemical structure of Evo. (**B**) HepG2 and SMMC-7721 cells were incubated with increasing concentrations of Evo (0, 0.25, 0.5, 1, 2, and 4 µM) for 24 h. Cell Counting Kit-8 (CCK-8) assay was performed to detect the cytotoxic effect of Evo. (**C**) Hoechst 33258 staining of HepG2 and SMMC-7721 cells after being treated with Evo (0, 0.5, and 1 µM) for 24 h. Apoptotic cells were identified by the presence of bright-blue fluorescent and highly condensed or fragmented nuclei (×40). (**D**) Flow cytometry histograms of cell apoptosis distribution after treatment with Evo (0, 0.5, and 1 µM) for 24 h by Annexin V-FITC/PI double staining. (**E**) Representative images from the colony-formation assay. HepG2 and SMMC-7721 cells were incubated with or without Evo (0, 0.5, and 1 µM) for 24 h and allowed to grow into colonies for another 16 days. Values are means and standard errors of three separate experiments (* *p* < 0.05 and ** *p* < 0.01 versus control).

Initially, we detected the anti-proliferation effect of Evo (Figure 1A) on HepG2 and SMMC-7721 cells. Cell viability was investigated after HepG2 and SMCC-7721 cells were treated with different concentrations (0, 0.25, 0.5, 1, 2, and 4 µM) of Evo for 24 h using the CCK-8 assay. As shown in Figure 1B, viability of HepG2 and SMMC-7721 cells was significantly reduced when treated with Evo for 24 h. Moreover, half maximal-inhibitory concentration (IC50) of Evo at 24 h for HepG2 and SMMC-7721 cells was approximately 1 µM. Thus, we used Evo at a concentration of 1 µM for subsequent experiments. Therefore, HepG2 and SMMC-7721 cells were treated with an absence or presence of Evo at concentrations of 0.5 and 1 µM of Evo for 24 h, cells were then stained with Hoechst 33258. Changes in nuclear morphology of Evo-exposed cells were observed under a fluorescence microscope and featured a marked increase in the quantity of apoptotic chromatin condensation and nuclear fragmentation (Figure 1C). Meanwhile, flow cytometry analysis revealed that the apoptotic rate of HepG2 and SMMC-7721 cells increased after being treated with different concentrations (0, 0.5, and 1 µM) of Evo for 24 h (Figure 1D). In addition, we assessed the effect of Evo (0, 0.5, and 1 µM) on colony formation of HepG2 and SMMC-7721 cells after 16 days and observed a significant and dose-dependent inhibition of colony formation with HepG2 and SMMC-7721 cells relative to untreated controls (Figure 1E). Taken together, these data suggest that the inhibitory effect of Evo on HepG2 and SMMC-7721 cell growth was associated with cell apoptosis.

### 2.2. Evo Decreases Expression of NOD1 and Results in Suppression of NF-κB and MAPK Activation In Vitro

**Figure 2:**
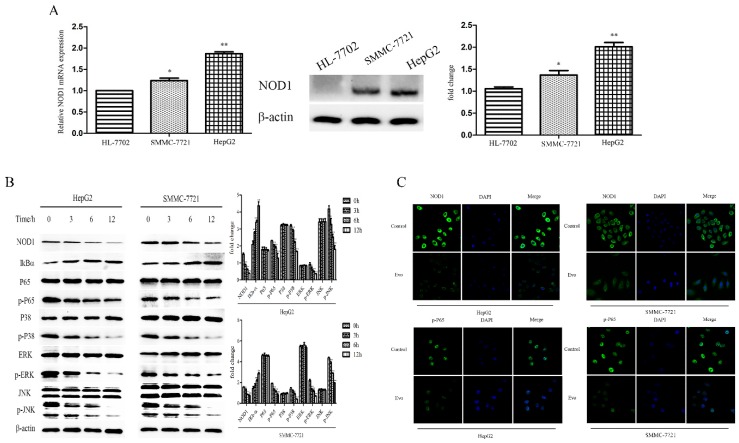
Evo decreases expression of NOD1 resulting in suppression of nuclear factor-κB (NF-κB) and mitogen-activated protein kinase (MAPK) activation in vitro. (**A**) Expression of NOD1 in normal hepatocyte HL-7702, HepG2, and SMMC-7721 cells were detected by qRT-PCR and Western blot assays. (**B**) HepG2 and SMMC-7721 cells were incubated with 1 µM Evo for 0, 3, 6, and 12 h. The Western blot assay was performed to detect levels of proteins in the NOD1 pathway. (**C**) Representative images from the immunofluorescence method (×400). HepG2 and SMMC-7721 cells were incubated with or without Evo (0, 0.5, and 1 µM) for 24 h to detect levels of NOD1 and p-P65. Values are means and standard errors of three separate experiments (* *p* < 0.05 and ** *p* < 0.01 versus control).

NOD1 levels of normal hepatocyte HL-7702, HepG2, and SMMC-7721 cells were detected by qRT-PCR and Western blot assays. As shown in Figure 2A, in contrast to that in HL-7702 cells, NOD1 levels were significantly increased in HepG2 and SMMC-7721 cells. To provide further insight we used a Western blot assay to detect levels of proteins in the NOD1 pathway on HepG2 and SMMC-7721 cells treated with 1 µM Evo for 0, 3, 6, and 12 h. We found NOD1, p-P65, p-ERK, p-p38, and p-JNK were decreased and IκBα was increased after Evo treatment in a time-dependent manner compared with control (untreated) cells (Figure 2B). Subsequently, we further investigated the expression of NOD1 and p-P65 in HepG2 and SMMC-7721 cells treated with 1 µM for 24 h, as analyzed by the immunofluorescence method. Compared to control (untreated) cells, Evo treatment showed significant decreases in NOD1 and p-P65 levels (Figure 2C). From these results, we concluded that NOD1 levels in HepG2 and SMMC-7721 cells exceeds that in the HL-7702 cells and that Evo decreases the expression of NOD1, resulting in the suppression of NF-κB and MAPK activation pathways.

### 2.3. Evo Induces Apoptotic Cell Death of HepG2 and SMMC-7721 Cells via the NOD1-Mediated Apoptotic Pathway In Vitro

**Figure 3:**
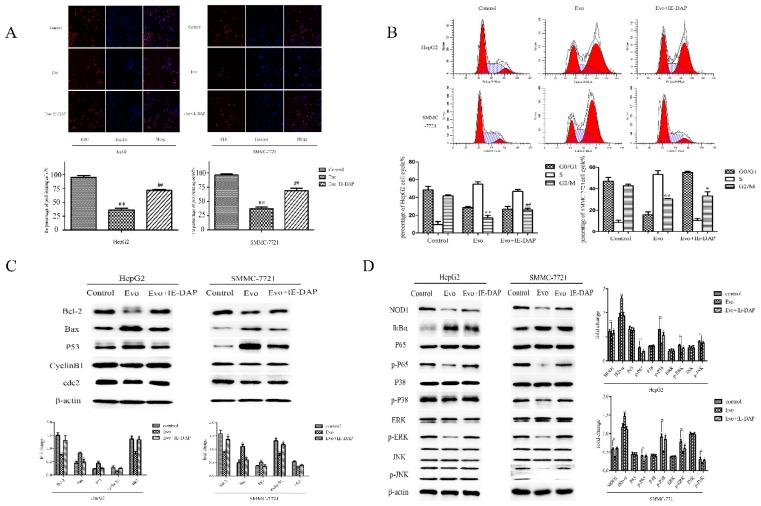
Evo induces apoptotic cell death of HepG2 and SMMC-7721 cells via the NOD1-mediated apoptotic pathway in vitro. HepG2 and SMMC-7721 cells were treated with 10 µg/mL IE-DAP for 2 h before exposure to 1 µM Evo. Apoptosis (**A**), cycle arrest (**B**), apoptosis-related protein levels (**C**) and the proteins’ levels of NOD1 pathway (**D**) in treated and untreated cells were measured by 5-ethynyl-2′-deoxyuridine (EdU) (×40), cellular propidium iodide (PI) fluorescence and Western blot methods. The percentage of proliferating cells (EdU+) was quantitated using ImageJ software (National Institutes of Health, Bethesda, MD, USA). Values are means and standard errors of three separate experiments (* *p* < 0.05 and ** *p* < 0.01 versus control, ^#^ *p* < 0.05 and ^##^ *p* < 0.01 versus Evo).

To further validate that Evo-induced apoptosis was mediated by the NOD1-dependent pathway, we used the specific agonist, IE-DAP (10 µg/mL), which specifically activates NOD1, to treat HepG2 and SMMC-7721 cells for 2 h before exposure to 1 µM Evo. Pre-treatment with IE-DAP rescued HepG2 and SMMC-7721 cells from Evo-induced suppression of proliferation measured by 5-ethynyl-2′-deoxyuridine (EdU) (Figure 3A). Cycle arrest was detected by cellular propidium iodide (PI) fluorescence (Figure 3B). The levels of apoptosis-related proteins and NOD1 pathway proteins were measured by Western blot methods (Figure 3C,D). Since the specific agonist for NOD1 attenuates Evo-induced apoptosis and cycle arrest, we concluded that Evo induces apoptotic cell death of HepG2 and SMMC-7721 cells via the NOD1-mediated apoptotic pathway.

### 2.4. Evo Inhibits HCC Cell Growth In Vivo

**Figure 4:**
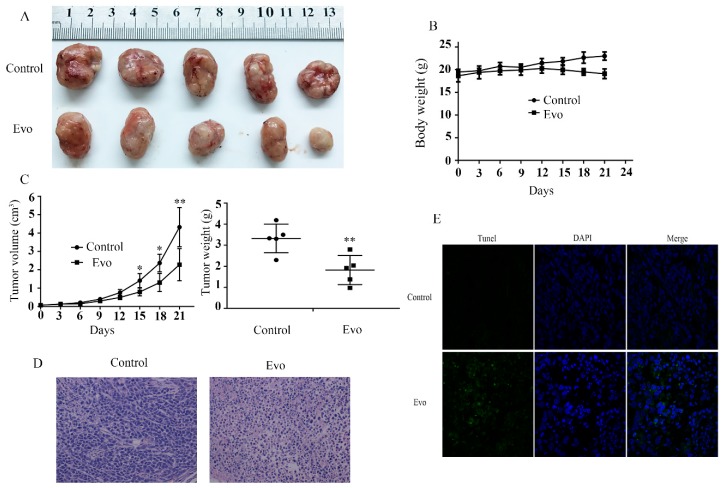
Evo inhibits HCC cells growth in vivo. (**A**) Representative images of the tumor generated by controls and Evo treatments. (**B**) Mice body weights were recorded and compared. (**C**) Tumor volumes were recorded and compared. Tumor weight was obtained at the end of the experiment when mice were sacrificed by dislocation after anesthesia. (**D**,**E**) Xenograft tumor tissues were analyzed for hematoxylin-eosin staining (HE) staining (×200) and TdT-mediated dUTP Nick-End Labeling (TUNEL) (×400). Values are means and standard errors of five separate experiments (* *p* < 0.05 and ** *p* < 0.01 versus control).

Following investigation of apoptosis in HCC cells in vitro, we further investigated whether Evo could inhibit HCC cell growth in vivo. Firstly, about 3 × 10^6^ HepG2 cells were injected at the right armpit of 6-week-old BALB/c nude mice to establish the tumor model. After 8–10 days when the tumors have formed, the experimental animals were treated with Evo at a 10 mg/kg dose. The mice were weighed every three days. At the end of three weeks the animals were sacrificed and the tumors were excised and weighed (Figure 4A). We initially explored the toxicity of Evo in mice. The control mice and those treated with Evo showed no significant difference in weight, which meant that Evo treatment did not cause significant systemic toxicity (Figure 4B). However, results shown in Figure 4C indicate that Evo treatment inhibited tumor growth in vivo, as evidenced by small tumor volumes and reduced tumor weights in Evo-treated mice. Moreover, histopathologically, hematoxylin-eosin staining (HE) staining and TdT-mediated dUTP Nick-End Labeling (TUNEL) results showed that there was a lot of cell necrosis and damage in experimental mice compared with control mice (Figure 4D,E). These results are the same to those seen in vitro.

### 2.5. Evo-Induced Apoptosis of HCC Cells Occurred via the NOD1 Pathway In Vivo

**Figure 5:**
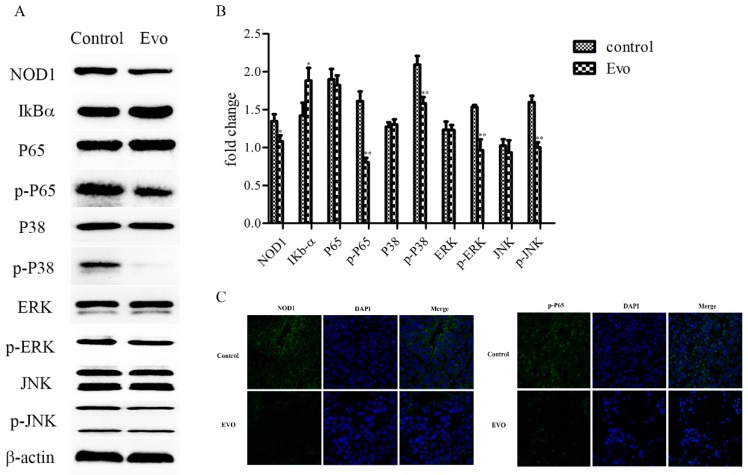
Evo-induced apoptosis of HCC cells occurred via the NOD1 pathway in vivo. (**A**,**B**) The mice treated with or without 10 mg/kg of Evo. Levels of proteins in the NOD1 pathway in tumor tissues were detected by the Western blot method. (**C**) Representative images from the immunofluorescence method. Expression of NOD1 (×400) and p-P65 (×200) in tumor tissues was analyzed by the immunofluorescence method. Values are means and standard errors of five separate experiments (* *p* < 0.05 and ** *p* < 0.01 versus control).

To confirm whether the inhibition of Evo on xenograft tumor growth is mediated by blocking the NOD1 pathway we detected the levels of NOD1, IκBα, p-P65, p-ERK, p-p38, and p-JNK proteins in xenografted tumors of mice treated with Evo by Western blot analysis. As shown in Figure 5A,B, levels of NOD1, p-P65, p-ERK, p-p38, and p-JNK proteins were decreased and the level of IκBα was increased in experimental mice compared with control mice. We analyzed tumors by the immunofluorescence method to investigate levels of NOD1 and p-P65. There were decreases in NOD1 and p-P65 levels in xenografted tumors of mice treated with Evo, which was consistent with the data in vitro (Figure 5C).

## 3. Discussion

HCC is the most frequent primary liver cancer and is an important medical problem. The majority of HCC cases are diagnosed at later stages and it is estimated that approximately half of HCC patients have poor prognosis and survival rates [31]. Therefore, the development of novel and less toxic therapeutic agents is imperative to reduce high morbidity and mortality rates associated with HCC. Over the years, the antitumor effect of natural products has been a key therapeutic strategy for treatment of cancers [32,33,34]. Alternation of oncogenes and tumor suppressor genes could induce transformation of normal cells and tumor cells in the process of tumorigenesis [35]. There are some keys element to regulate apoptosis, these include the apoptotic-inducing proteins Bax and P53 and the anti-apoptotic protein Bcl-2. Functional failure of anti-apoptotic proteins causes the loss of cell function and release of apoptotic-inducing factors and eventual cell apoptosis [36]. Researchers have found Parthenolide upregulated Bax and P53 proteins and downregulated Bcl-2 to inhibit the proliferating effect of nicotine on lung cancer [37]. Coptisine has reported to induce apoptosis in HCT-116 cells by upregulating Bax and downregulating Bcl-2 [38].

*Evodia rutaecarpa Bentham* is a traditional herbal medicine commonly used in Asia as an analgesic, anti-emetic, hemostatic, anti-hypertensive, and uterotonic agent [39]. Evodiamine, an active ingredient isolated from the fruit of *Evodia rutaecarpa Bentham*, has been well documented to inhibit the proliferation of various human cancer cells [15,16,17,18,19,20,21,22,23,24]. Previous study has shown that Evo could also change the morphology and decrease viability and proliferation of renal carcinoma cells [40]. Additional research reported that Evo could induce apoptosis of human leukemia HL-60 cells by upregulating Bax and P53 proteins and downregulating Bcl-2 [41]. Evo inhibited growth of human colorectal carcinoma cells by increasing expression of Bax and p53 and decreasing expression of Bcl-2 [42]. Evo induced G2/M cell cycle arrest and decreased expression of CyclinB1 and cdc2 in lung cancer [16]. Evo also induced G2/M arrest and apoptosis in H446 and H1688 human small-cell lung cancer cells [43]. In our study, we proved the effects of Evo on HepG2 and SMMC-7721 cells. Our results indicate that Evo inhibited cell proliferation and led to cell cycle arrest at the G2/M phase. This effect appears to be due to downregulation of Bcl-2, CyclinB1, and cdc2 and upregulation of Bax and P53. The same results were also seen in vivo.

It has been reported that Evo exerts antitumor activities by suppressing NF-κB and MAPK activation [15,20,25]. NF-κB and MAPK participate in many biological functions such as cell survival, proliferation, migration, invasion, and apoptosis [44]. In addition, recent research found that NOD1 (in the NOD1 pathway), a class of pattern-recognition-receptors (PRRs) characterized by three motifs containing a C-terminal leucine-rich repeat (LRR) domain, a central nucleotide-binding domain (NBD), and variable N-terminal caspase recruitment domains (CARD), could initiate NF-κB-dependent and MAPK-dependent gene transcription [26]. NOD1 are expressed in most tissues, including cancer cells. From gathering data of NOD1 levels in the GEO database, researchers revealed that NOD1 expression differed significantly between tumor and non-tumor tissue [26]. Recent experimental studies have also reported that the NOD1 pathway is related to controlling development of various tumors [27,28,29,30]. In our study, results revealed that NOD1 levels were significantly increased in HCC cells compared with normal hepatocyte cells. Thus, we hypothesize that development of HCC cells is related to the expression of NOD1. We also found that Evo altered the levels of proteins in the NOD1 pathway, which indicates that Evo decreases the expression of NOD1, resulting in suppression of NF-κB and MAPK activation. Some studies have shown that the sodium iodide symporter is involved in tumor progression by activating NF-κB and MAPK [45]. Phenethyl Isothiocyanate (PEITC) and Benzyl Isothiocyanate (BITC) inhibited cell migration and invasion of Human Melanoma cells by affecting activation of MAPK [46]. Brusatol inhibited growth and induced apoptosis in pancreatic cancer by affecting activation of JNK, p38 MAPK, and NF-κB [47]. Based on these observations, our results show that IE-DAP (the specific agonist of NOD1) could weaken the effect of Evo on the suppression of activating NF-κB and MAPK and Evo-induced apoptosis of HCC cells. These findings suggest that Evo exerted anti-hepatocellular carcinoma activity maybe via the NOD1 pathway.

**Figure 6:**
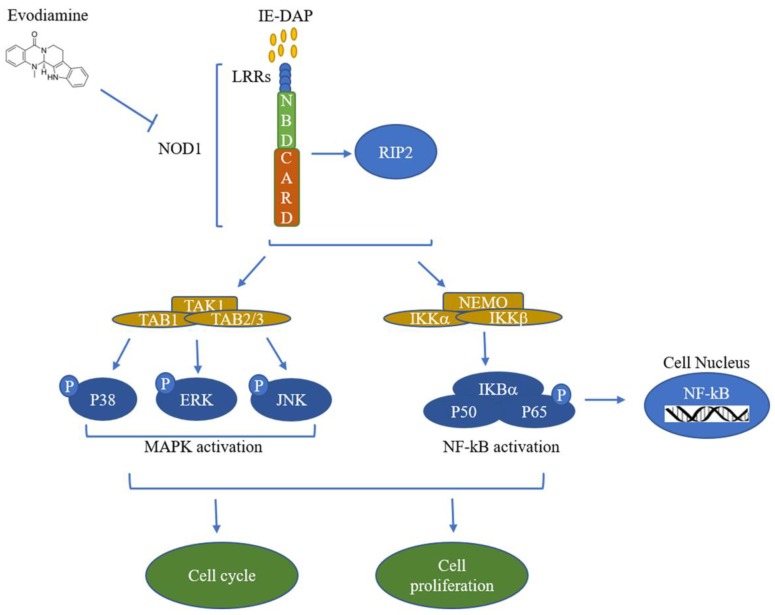
Model of NOD1 signaling cascade. Nucleotide-binding oligomerization domain 1 (NOD1) characterized by three motifs containing a C-terminal leucine-rich repeat (LRR) domain, a central nucleotide-binding domain (NBD), and a variable N-terminal caspase recruitment domains (CARD), which could be activated by γ-D-Glu-mDAP (IE-DAP). NOD1 recruits the adaptor RIP2 and facilitates recruitment of TAK1 and NEMO. Here the TAK1 recruits TAB1 and TAB2/3 inducing MAPK activation. In addition, NEMO instigates activation of NF-κB by reducing IκBα and phosphorylating P65, the subunit of NF-κB. Furthermore, activation of MAPK and NF-κB promotes the cell cycle and proliferation. In our study, Evodiamine could potentially suppress the NOD1 signal pathway to inhibit the cell cycle and proliferation. The arrows represents the activation and the T bar represents the suppression.

In summary, our results demonstrate that Evo could induce apoptosis remarkably and the inhibitory of Evo on HCC cells may be through suppressing the NOD1 signal pathway in vitro and in vivo. (Figure 6).

## 4. Materials and Methods

### 4.1. Reagents and Antibodies

Evo was purchased from Nanjing Zelang Pharmaceutical and Biological Products Company (Nanjing, China). A 10 mM solution of Evo was prepared in dimethyl sulfoxide (DMSO) and stored at −20 °C. Antibody against NOD1 (NB100-56152) was purchased from Novus Biologicals (San Diego, CA, USA). Antibodies against IκBα (sc-203), P38 (sc-7972), p-P38 (sc-17852-R), ERK (sc-94), p-ERK1/2 (sc-16982), JNK (sc-571), p-JNK (sc-6254), Bcl-2 (sc-509), Bax (sc-20067), and P53 (sc-47698) were purchased from Santa Cruz Biotechnology (Santa Cruz, CA, USA). Antibodies against P65 (D14E12), p-P65(ser-536), cyclin B1 (D5C10), cdc2 (E1Z6R), and β-actin (8H10D10) were purchased from Cell Signaling Technology (Danvers, 3 Trask Lane, USA). The NOD1 agonist IE-DAP was purchased from InvivoGen (San Diego, USA). Cell Counting Kit-8 was purchased from Dojindo (Kumamoto, Japan). Hoechst 33258 was purchased from Beyotime Institute of Biotechnology (Shanghai, China).

### 4.2. Cell Culture and Culture Condition

Normal hepatocyte HL-7702 and hepatocellular cancer cells HepG2 and SMMC-7721 were all a gift from the Gene Research Division of the Third Military Medical University Institute, Chongqing, China. Cells were cultured with Dulbecco’s Modified Eagle Medium (DMEM, Gibco-Invitrogen Corporation, Carlsbad, CA, USA) and 10% fetal bovine serum (FBS, Gibco-Invitrogen Corporation, Carlsbad, CA, USA) and 1% antibiotic (Beyotime Institute of Biotechnology, Shanghai, China) in a humidified atmosphere with 5% CO_2_ at 37 °C.

### 4.3. Cell Viability Assay

Cell viability was determined using Cell Counting Kit-8 (CCK-8) [48]. Briefly, according to the manufacturer’s instructions, cells were plated in 96-well plates at a density of 6 × 10^3^ cells per well. After attachment for 24 h, the medium was changed for fresh medium containing Evo with indicated or different concentrations respectively and cells were incubated for another 24 h. After indicated cultivation time, the viability of cells was measured using the CCK-8 assay. Before testing, CCK-8 solution (10 µL) was added to each well containing a 100 µL mixture of culture medium. The plates were incubated for 2 h at 37 °C in the incubator. Cell viability was counted by absorbance measurements at 450 nm using an auto microplate reader (Bio-Rad, Hercules, CA, USA). The OD_450_ value was proportional to viability of the cell. All experiments were performed in triplicate.

### 4.4. Hoechst 33258 Staining

Hoechst 33258 staining (Beyotime Institute of Biotechnology, Shanghai, China) was used for the detection of morphological apoptosis [49]. After being treated with Evo (0, 0.5, 1 µM) for 24 h, HepG2 and SMMC-7721 cells were washed with PBS (phosphate buffered solution) and fixed 4% paraformaldehyde for 30 min at room temperature. Cells were washed with PBS twice and stained according to manufacturer’s instructions for 15 min. Alterations in morphology of the nuclei were observed using fluorescence microscopy (Olympus, Japan). Cells were designated as apoptotic cells based on nuclear morphology changes, such as bright-blue fluorescent and condensed nuclei.

### 4.5. Flow Cytometric Analysis

HepG2 and SMMC-7721 cells were seeded at a density of 2 × 10^5^/mL in six-well plates. For apoptosis rate analysis, after 24 h cells were exposed to Evo with different concentrations (0, 0.5, 1 µM) for another 24 h. Cells were then collected and washed twice with PBS to remove the medium. At least 1 × 10^5^ cells were resuspended in 100 µL binding buffer containing Annexin V-FITC/propidium iodide (PI) according to the manufacturer’s protocol [50] (Beyotime Institute of Biotechnology, Shanghai, China). Cell cycle analysis: after 24 h, we used IE-DAP (10 µg/mL) to treat HepG2 and SMMC-7721 cells for 2 h before exposure to 1 µM Evo for another 24 h. Cells were collected and fixed in 70% ethanol for 24 h at 4 °C, the cell pellet was resuspended in PBS (400 µL), RNaseA (10 mg/mL, 50 µL), and Propidium iodide (PI) (2mg/mL, 10 µL). Mixtures were incubated in the dark at 37 °C for 30 min. Then cell apoptosis rates and cycle were analyzed with FAC-Scan laser flow cytometry (FAC-S, Calibur, Becton Dickinson, Franklin Lakes, NJ, USA). Data was analyzed using CELL Quest software (1.1 version, BD Biosciences, Franklin Lakes, NJ, USA).

### 4.6. Colony-Formation Assay

HepG2 and SMMC-7721 cells were plated in plates of 60 mm diameter at a density of 2.5 × 10^2^ and treated with or without Evo for 24 h. Cells were incubated for another 16 days in Evo-free conditions. Colonies were fixed with 4% paraformaldehyde for 30 min at room temperature. The cells were washed with PBS twice and stained with Wright-Giemsa solution (Beyotime Institute of Biotechnology, Shanghai, China). Plates were photographed and the number of colonies was calculated [51]. Results represented the average of 3 independent experiments.

### 4.7. Reverse Transcription and Real-Time PCR Assay

Total RNA was isolated from cultured cells by homogenization in Trizol reagent (Gibco-Invitrogen Corporation, Carlsbad, CA, USA), used for RNA extraction. RNase-free DNase I was used to remove contaminated DNA. The quality of RNA was measured using a NanoDrop 2000 Spectrophotometer (Thermo Fisher Scientific Inc., Waltham, MA, USA). 1 µg total RNA was reverse transcribed into the first-strand cDNA via PrimeScript RT reagent kit (TaKaRa Biotech. Co., Tokyo, Japan). The mRNA expression of NOD1 was analyzed with respect to the housekeeping gene β-actin in a CFX ConnectTM Real-Time PCR detection system (Bio-Rad, Hercules, CA, USA) at the following cycling conditions: 95 °C for 2 min, followed by 40 cycles of 95 °C for 5s, and 60 °C for 30 s. The melting curve was also done from 65 to 95 °C with 0.5 °C·s^−1^ increments to exclude unspecific amplification after the last reaction. The relative expression levels in terms of fold changes of target genes were calculated by 2^−∆∆*C*T^ method [52]. The primers (Sangon Biotech, Shanghai, China) were as follows: NOD1 forward primer 5′-ACTGAAAAGCAATCGGGAACTT-3′, NOD1 reverse primer 5′-CACACACAATCTCCGCATCTT-3′; β-actin forward primer 5′-GTACCACTGGCATCGTGATGGGACT-3′, β-actin reverse primer 5′-CCGCTCATTGCCAATGGTGAT-3′.

### 4.8. Immunoblot Assay

Cells were isolated and washed with cold PBS and resuspended in lysis buffer containing 1% Phenylmethanesulfonyl fluoride and phosphatase inhibitor added just before use (Beyotime Institute of Biotechnology, Shanghai, China). After incubation for 30 min on ice, the supernatant was collected by centrifugation at 12,000 rpm for 15 min at 4 °C and the protein concentration was determined by BCA Protein Assay Kit (Beyotime Institute of Biotechnology, Shanghai, China). Equal amounts of proteins from each sample were separated by sodium dodecyl sulfate-polyacrylamide gel electrophoresis (SDS-PAGE) and separated proteins were transferred to polyvinylidene difluoride (PVDF) membranes. The membranes were blocked with 5% non-fat milk for 2 h, incubated with primary antibody overnight at 4 °C, washed in Tris-buffered saline with Tween20 (TBST) for 30 min, and incubated with IgG horseradish peroxidase (HRP)-conjugated secondary antibody for 1.5 h at room temperature. Bound immune-complexes were detected by enhanced chemiluminescence (ECL) Western blotting detection reagents (Millipore, 8 Valencia Street, Belize, USA) and exposure to the Luminescent Image Analyzer (Bio-Rad, CA, USA) [53].

### 4.9. Immunofluorescence Staining

The 2 × 10^4^ cells were seeded in six-well plates, put in a glass slide for 24 h, and treated with or without Evo for 24 h. Cells were then fixed in 4% paraformaldehyde for 30 min at room temperature and washed twice for 10 min followed by permeabilization with 0.5% Triton X-100 in PBS for 20 min. Fixed cells were blocked with 1% goat serum albumin for 1 h, then incubated with primary antibody against NOD1, p-P65 at 4 °C overnight. After being washed thrice with PBS, cells were stained with FITC-conjugated secondary antibodies (GeneTex, Irvine, CA, USA) at 37 °C for 2 h. Nuclei were stained with 4′,6-diamidino-2-phenylindole (DAPI) for the last 5 min. Images were captured by fluorescence microscope (Olympus, Japan) [54].

### 4.10. EdU Proliferation Assay

Cells were seeded in 96-well plates. Cells were incubated under standard conditions in complete media overnight. After 24 h, we treated (with or without IE-DAP (10 µg/mL)), the HepG2 and SMMC-7721 cells for 2 h, before exposure to 1 µM Evo for another 24 h. Cell proliferation was detected using the incorporation of 5-ethynyl-2′-deoxyuridine (EdU) with the EdU Cell Proliferation Assay Kit (Ribobio, Guangzhou, China). Briefly, cells were incubated with 50 µM EdU for 6 h before fixation, permeabilization, and EdU staining, which were performed according to the manufacturer′s protocol. Cell nuclei were stained with DAPI for 20 min. The proportion of cells incorporated with EdU was determined with a fluorescence microscope [55].

### 4.11. Tumor Xenograft Models in Nude Mice

All procedures involving mice and experimental protocols were approved by the Laboratory Animal Center of Chongqing Medical University. BALB/c nude mice, 6-weeks-old, were purchased from the Laboratory Animal Center of Chongqing Medical University. HepG2 cells (3 × 10^6^ cells in 0.2mL of PBS) were subcutaneously injected at the right armpit of nude mice as previously described. After 8–10 days when tumors have formed, the experimental animals were treated with Evo at a dose of 10 mg/kg. The mice were weighed and tumors measured with a caliper every three days. Tumor volume (cm^3^) was calculated by the formula: Volume = (width)^2^ × length/2 to present the tumor growth curve. At the end of the experiment, all animals were sacrificed by dislocation after anesthesia (Sigma, St. Louis, MO, USA) [56] and xenograft tumors were collected and measured by HE staining (Millipore, 8 Valencia Street, Belize, USA). This calculated the different cellular morphology and TUNEL (Millipore, 8 Valencia Street, Belize, USA), calculated as the product of intensity of staining and percentage of positively stained cells.

### 4.12. Statistical Analysis

Figures in the text are representative of at least three independent experiments. The data is expressed as means ± SD (standard deviation). Significance of the differences between various experimental and control groups were statistically analyzed using Student’s t-test with SPSS 22.0 software (IBM, 1 New Orchard Road, Armonk, New York, USA) and indicated as * *p* < 0.05, ** *p* < 0.01.

## 5. Conclusions

In this study, Evo can inhibit proliferation and induce apoptosis in HCC cells. Furthermore, the effects of Evo may be achieved through inhibiting the NOD1 signal pathway. We confirmed that Evo had an anti-tumor effect in HepG2 and SMMC-7721 cells, possibly via suppressing the NOD1 pathway, both in vitro and in vivo. Therefore, these findings provide support for further investigation of Evo as a therapeutic agent for HCC.

## Abbreviations

HCC	hepatocellular cancer
Evo	Evodiamine
DAMPs	danger-associated molecular patterns
PRRs	pattern recognition receptors
NOD1	Nucleotide-Binding Oligomerization Domain 1
LRR	leucine-rich repeat
NBD	nucleotide-binding domain
CARD	caspase recruitment domains
NF-κB	nuclear factor-κB
MAPK	mitogen-activated protein kinase
